# Reproductive practices by patterns of marriage among Iranian women: study protocol for an explanatory sequential mixed methods design

**DOI:** 10.1186/s12978-015-0080-1

**Published:** 2015-09-18

**Authors:** Ziba Taghizadeh, Abouali Vedadhir, Fereshteh Behmanesh, Abbas Ebadi, Abulghasem Pourreza, Mohammad Jalal Abbasi-Shavazi

**Affiliations:** 1 Department of Midwifery and Reproductive Health, School of Nursing and Midwifery, Tehran University of Medical Sciences, Tehran, Iran; 2 Department of Anthropology, Faculty of Social Sciences, University of Tehran, Tehran, 14117-13118 Iran; 3 HRA, UCL Department of Science and Technology Studies, University College London, Gower Street, London, WC1E 6BT UK; 4 PhD Candidate in Reproductive Health, School of Nursing and Midwifery, Tehran University of Medical Sciences, Tehran, Iran; 5 Behavioral Sciences Research Center (BSRC) and Faculty of Nursing, Baqiyatallah University of Medical Sciences, Tehran, Iran; 6 Department of Management and Health Economics, School of Public Health, Tehran University of Medical Sciences, Tehran, Iran; 7 Department of Demography, Faculty of Social Sciences, University of Tehran, Tehran, Iran; 8 Crawford School of Public Policy, Australian National University Canberra, Canberra, Australia

**Keywords:** Patterns of marriage, Reproductive practices, Study protocol, Mixed methods design

## Abstract

**Background:**

Nowadays, nearly half of the world population lives in societies with low fertility or the below-replacement fertility. This potentially grounds the critical situation of reduction in the workforce and causes the aging of population due to an overall increase in life expectancy and standard of living. Hence, population and its transitions including the issue of fertility decline has become a topic of intense debate in the agenda-setting and policy-making processes in both the developed and developing countries. In this view, what can practically be done to respond to the fertility decline that entails effectively addressing the determinants of fertility change? In line with the literature, how people form their marriages or patterns of marriage is amongst influencing factors which potentially affect their reproductive practices as diverse societies recognize different conventions for marriage. This study is to examine women’s reproductive practices by the various patterns of marriage using the explanatory sequential mixed methods design.

**Methods/design:**

This study has an explanatory sequential mixed methods design, the follow-up explanations variant model, with two strands. This design will be implemented in two distinct phases. In the first phase, a cross-sectional quantitative study will be done using a cluster sampling strategy on 850 married women 15–49 years old living in Babol city, Iran. In order to obtain a deeper understanding of the results of the quantitative phase, researchers will implement a qualitative research in the second phase of this study. This design will provide an explanation of the quantitative research results using the qualitative evidence.

**Discussion:**

As patterns of marriage have implications for the status of women, their health and fertility, the result of this study can provide a rich source of information for the required health-related interventions and policies are required to put the demographic changes on the right track at micro and macro level and improve the reproductive practices of women at micro level.

## Background

Fertility refers to the actual production of offspring or the number of children born alive per woman. Fertility goes beyond its biomedical aspect as it has various socio-cultural, emotional, cognitive and political aspects, being subject to change in different societies around the world, based on various factors. Status of women and their motivation for childbearing among populations and sub-populations vary from society to society and this brings about the average number of children from women of different ethno-cultural groups to be unequal [1].

A closer look at the theoretical models in demography reveals that most of these models have defined fertility as the result of rational decision-making process in which the costs and benefits of any action are measured. For instance, the theory of planned behavior (TPB) is one of these models. This model suggests that the behavior of each individual stems from their intentions, which are actually the result of subjective norms and attitudes. People’s attitudes reflect their personal beliefs and values and subjective norms reflect a set of issues that are important for others. People give credence to the attitudes and norms to form intent, which is a key determinant of behavior [2, 3]. Human behavior is a function of social and cultural environment [4] and individual and cultural differences that exist between individuals and communities cause different reproductive behavior [2].

In recent years, different family planning strategies were used to manage population and control birth as a result of a number of problems or crises posed by increasing population in different communities. However, over the past few decades, population-related problems and concerns have not been limited to the over-population and rapid growth of population, fall in fertility, likewise, they can become a problem or subject for concern. This issue has quickly spread all over the world and now nearly half the world’s population lives in countries with low fertility or the below-replacement level. It is anticipated that in the mid-21st century, in three-quarters of the developing countries, the fertility will reach to replacement level or below this level [5]. During the last decades of the previous century, throughout Europe the fall in birth rate was such that it led to the invention of a new term, lowest-low fertility for total fertility rate of 1.3 or Ultra-low for total fertility rate of 1 [6] and a significant decrease in the population of Europe was predicted [7].

Many countries in South and Southeast Asia have completed their demographic transition including the age structural transition. In these countries fertility rate is in the replacement level [8]. A closer look at the fertility rate in Iran reveals that the below-replacement level of fertility is widespread in contemporary Iran [9]. With fertility in the replacement level or the below-replacement level, the relative portion of population under 15 years is reduced, while due to increased life expectancy and mortality at older ages, the proportion of the population aged 65 and over is increased [10, 11]. This age-structure transition potentially causes the critical situation of reduction in the workforce and the aging of population due to an overall increase in life expectancy [12]. Hence, population and its transitions including the issue of fertility decline has become a topic of intense debate in the agenda-setting and policy-making processes in both developed and developing countries including in the context of the Islamic Republic of Iran.

The experience and evidence of fertility transition in Iran indicate that, notwithstanding the effectiveness of the family planning policies in rapidly decreasing fertility rate which has continued from the second half of the 1980s, the changes in people’s reproductive practices are influenced by other factors. In fact, the process of fertility transition in Iran is considered proportionate to the changes occurred in various economic, social and some traditional family aspects, leading to the related changes in patterns of marriage and, ultimately, changes in behavior, ideals and aspirations of the people for childbearing [12, 13].

In the past, due to the influence of kinship and strengthening ethnical solidarity, the process of marriage was arranged by the significant others including parents. Nowadays, it seems that the role and autonomy of individual (offspring) in marriage and selecting their spouse has significantly improved and the pattern of marriage has changed from a traditional one, mostly arranged and selected by parents, to a modern and even post-modern ones, mostly based on the romantic relationships processes [8, 14–19]. Pattern of marriage can also include any combination of all of the above patterns. In this view, patterns of marriage refer to the ways of commencing a relationship, leading to and arranging a marital relationship and eventually forming a family. Some studies revealed that the increasing autonomy of women to choose their husbands has increased their role in household decision-making and also increased their decision-making power in the fertility-related relations and proper number of children. This is one reason that these years led to the population decline [20]. Therefore, prior to implementing any policy and action in the field of fertility and population, the causes or reasoning of reproductive behaviors [or in other words, reproductive practices] such as low fertility should be examined [11–13, 21]. In this view, reproductive practices don’t take place in a vacuum, the socio-cultural and political context and structure of family and how it has formed can affect these practices at multiple levels. Up till now, however, the role of marriage patterns in fertility changes and practices have been less studied, and which interventions at the household level can slow down or reverse the fertility decline have been heavily debated [7].

Since the patterns of marriage may affect the reproductive practices which are central to the sexual and reproductive health, an examination of these practices by monitoring the changing patterns of marriage can be valuable for meeting the reproductive and sexual health needs of women. These practices are also related to the women’s health and preserving women’s health is not only a basic human right, but is also essential for the health of all nations. Women’s health affects long-term health of theirs, their family members and community [22]. knowing reproductive practices of women in traditional, modern, and even post-modern patterns of marriage is considered very important in making and implementing policy in all demographic, economic, political, socio-cultural and educational domains.

On the other hand, it seems patterns of marriage and reproductive practices interact with each other and probably, the most suitable method of this interaction is taking them into account and evaluating both of them simultaneously. Quantitative research results alone, without qualitative research, cannot lead to effective planning and interventions in women’s reproductive practices, and qualitative research on its own is not sufficiently effective to be used in operational planning due to its lack of generalization to the whole community. While using a mixed methods (MMs) approach can combine the strengths of quantitative studies (large sample size, generalization) and qualitative studies (listening to the participants, in-depth elaboration) and provide a more complete picture of the subject of study [23]. Hence, this MMs study was designed to address the following research objectives:

### Quantitative general purpose

To determine and compare women’s reproductive practices over patterns of marriage.

*Moreover, specific goals of the quantitative phase of this MMs study are as follows:*

1. Determine and compare the mean of the interval between marriage and first pregnancy over patterns of marriage.
2. Determine and compare the mean of child number over patterns of marriage.
3. Determine and compare the mean of birth interval over patterns of marriage.
4. Determine and compare the rate of abortion over patterns of marriage.
5. Determine and compare the rate of unwanted pregnancy over patterns of marriage.
6. Determine and compare the rate of contraceptive use before first pregnancy over patterns of marriage.

### Qualitative general purpose

To explain women’s reproductive practices over the patterns of marriage.

### General purpose of the MMs

To explain and interpret women’s reproductive practices over the patterns of marriage.

## Materials/design

This study has an explanatory sequential mixed methods (MMs) design, with the follow-up explanations variant model, valuing and integrating both objective and subjective data and results. This two-stage MMs research design will be carried out with the aim of interpreting the evidence of quantitative research from a study of women’s reproductive practices by different patterns of marriage in Babol city of Iran. In the first phase of the study the questionnaire of “women’s reproductive practices” will be used to gather quantitative data. After statistically analyzing the quantitative data, the second phase of the study, namely the qualitative stage, will be designed and carried out for the purpose of interpreting and explaining the results of the quantitative phase. This model typically starts with the data collection and analysis of a quantitative phase which is then followed by the qualitative research. This type of MMs study is designed and used when qualitative data is valuable for explaining or expanding the quantitative findings, particularly in the context where further explanation is merited concerning significant or non-significant differences among groups or patterns (e.g. patterns of marriage), key significant predictors and moderators, distinguishing demographic characteristics, surprising associations or contingencies and correlations, or unexpected results and outliers. In fact, the researchers give priority to the primary quantitative stage and the subsequent qualitative phase will help to explain the quantitative results [24, 25].

The sequence and weight of quantitative and qualitative of this MMs study can be visually presented using the notation system as follows: ***QUAN → qual***. This notation indicates that the study consists of two stages. The first stage is a quantitative research that has greater ability to achieve the objectives of the study, and the second one is a qualitative research conducted to explain the results of the first stage. Finally, an interpretation of how to explain the quantitative results using qualitative findings will be presented.

### Phase one: quantitative study

This phase is designed as a population-based cross-sectional study to examine the reproductive practices by patterns of marriage and some other socio-demographic characteristics using the “women’s reproductive practices” questionnaire. The questionnaire will be designed by the research team of this study by reviewing and upgrading “the fertility changes questionnaire in Iran” which was formerly developed by one of the authors of this paper (MJA-S). Then, the psychometric properties of the upgraded instrument will be, for a second time, evaluated in the setting of this study. To determine the patterns of marriage, the researchers will carry out a systematic review of literature. Then, they will design a number of items or questions regarding these patterns in the context of Iran by consulting with a panel of Iranian experts. The purpose of the review of literature is to provide the results to the members of the panel, prior to the meeting, in order to be more familiar with the existing patterns of marriage recognized in the literature. After this stage, the psychometric evaluation of questionnaire will be carried out.

#### Psychometric evaluation

The content validity (both qualitative and quantitative methods) and face validity (both qualitative and quantitative methods) will be performed in order to assess the validity of tools. In this way, first, the questionnaire will be sent to several experts or key informants (at least 10) to determine the qualitative content validity. The results will be studied in detail and the correction and editing will be done. Then, the questionnaire will be reviewed for content validity in terms of two indices: content validity ratio (CVR) and content validity index (CVI). To determine the CVR, the questionnaire will be sent to several experts (at least 10) and rated responses will be calculated by the formula. The results will be compared with the Lawshe’s Table and some questions may be removed. The amount of CVI will be determined using the method developed by Waltz and Bausell [26]. In this way, the three criteria of simplicity, specificity and clarity will be separately evaluated on a Likert scale by several experts (at least 10). In general, the inclusion and exclusion criterion of items in quantitative content validity will be as follows: the CVI items higher than 0.79 will be kept; Items with CVI between 0.70 and 0.79 will be modified, reviewed and corrected. The research team will try to remove items with CVI lower than 0.70. In the qualitative face validity phase, questions and items of the questionnaire will be examined regarding the level of difficulty, and the proportion and ambiguity, ease of completing the questionnaire, legibility, ease of understanding, grammar, style and spelling by face to face interviews with several married women from Babol. In the phase of quantitative face validity, several married women from Babol will comment on each question or item by using a Likert scale as follows: very important (5 points), important (4 points), relatively important (3 points), slightly important (2 points), and not important (1 point). Then, Impact score will be calculated using the related formula. In this phase, the questions with scores below 1.5 will be removed, and the rest of the questions and items that had scores higher than 1.5 will remain. The Test-re-Test methods with at least 20 individuals within 2 weeks and examined correlations will be used to assess reliability. Finally, a pilot study with at least 10 % of the primary sample size (n ≈ 850) will be conducted to determine the accurate size of sample.

#### Setting

The source population of this phase is every woman of reproductive age (15–49 years) meeting the inclusion criteria of this study and living in Babol, a city in Mazandaran province of Iran. Iran is one of the most ethno-culturally diverse societies in the world and the mosaic of peoples living in Iran today forms a unique situation of the country. The largest ethnic groups of Iran are Persian, Azerbaijani’s Turks, Kurds, Arabs, Baluchis, Lors, Mazandarani, Turkmen, Qashqai, Talysh and Gilaki. The north of Iran, in particular Mazandaran province, is the homeland for Mazandarani people. The population of Mazanderani people is about four million and the prevailing religion among this ethnic group is Shiite Islam [27, 28]. Babol, with a population of 512,944 people (2014), is one of the oldest and most important cities in the North of Iran, and women living in Babol can be a good representative for all Mazandarani women.

#### Sample size and sampling strategy

Out of all women of reproductive age (*N =* 166,183), approximate sample size in this phase of the study is 850. The exact size of sample will be determined, however, after evaluating psychometrics of the instrument and doing pilot study using at least 10 % of the approximate sample size. Strategy of sampling at this phase would be the multi-stage cluster sampling method. To do this, proportional random multistage cluster sampling is done in the 4 districts of Babol city, namely the North, the South, the East and the West out of 6 districts of the city. After choosing 4 districts, all villages, neighborhoods and blocks in each district will be randomly determined using the official information of the health center of Babol city. Next, the number of households in each cluster will be divided into four districts and the result will be multiplied by the total number of samples (n ≈ 850). The number of samples in each cluster will be achieved using the probability proportional to size (PPS) strategy. Thus, households are proportionally chosen from each block, and a woman of reproductive age (15–49 years) is chosen from these households. After determining the number of samples in each village and neighborhood and district, interviewers will be present at the clinic in the chosen village and the neighborhood with the permission of the health center of Babol city to complete the questionnaire. Indeed, the researchers will use a MMs strategy of sampling in this phase of study, as detailed by Teddlie and Yu [25]. Random probability sampling will be used to select the samples in first and second stages of sampling and the convenience sampling in the last cluster, i.e. in clinics.

Before starting the data collection in this phase, the informed consent will be obtained from all eligible Baboli women to assure ethical consideration or quality of the study. The Inclusion criteria of this phase of the study are as follows: Iranian nationality; able to speak in Persian or Mazandarani tongue; resident of Babol; being married women of reproductive age; having at least one child and with no diseases or deficiencies regarding reproduction such as the primary and secondary infertility; a kind of disease that can causes interference in their fertility; and no diagnosed severe mental disability and psychiatric disorders which may cause inability to respond to the questionnaire. The exclusion criteria of this study will be the lack of response to a maximum 10 % of the questions [29].

Participation will be voluntary and the participants will be free to refuse to fill out the questionnaire at any stage of the proceeding. Women’s phone numbers will be taken if they choose to participate in the qualitative phase of the study. Interviewers will ethically guarantee confidentially of their information.

#### Scales and data collection

The questionnaire comprises three sections, namely questions on the socio-demographic characteristics of women, the determinants of fertility including the patterns of marriage and the main reproductive practices. These sections are:

1. Socio-demographic details of the participants including age, education, place of birth, current place of living, current employment status (participant and her husband), socio-economic position of the family, number of marriages, age at first marriage, employment status before the first pregnancy, living with or without the family (first 2 years of marriage), existence of family relations before marriage (participant).
2. Patterns of marriage: As Rattray and Jones [30] suggested, one of the effective ways to design a questionnaire is the use of the panel of experts. Hence, in this stage of the study, key informants and professionals with diverse academic backgrounds will be requested to address and validate items relevant to the patterns of marriage in the socio-cultural and political context of contemporary Iran.
3. Reproductive practices measures: This questionnaire will be developed by the research team of this study by reviewing and adjusting the existing questionnaire of “Fertility changes in Iran” which was originally developed and used by one of the authors of this paper (MJA-S) to assess the reproductive behaviors of Iranian women about one decade ago [10].

#### Dependent and independent variables

In this study, the independent variable is the pattern of marriage and the dependent variables are reproductive practices. This study also has some socio-demographic variables in their capacity as confounding factors.

#### Data analysis

Data of the quantitative phase are managed and analyzed using the IBM SPSS software, v. 21. The exploratory data analysis (EDA) and basic descriptive and exploratory statistics including the mean, standard deviation, and frequency are performed for analyzing data sets to summarize their main characteristics and uncover the data recording errors often using both visual and statistical methods including frequency, percentage, mean, trimmed mean, m-estimators, median, smoothers, extreme values and outliers and standard deviation and the assumptions checking. These primary analyses are for understanding what the data can tell us beyond the formal modeling or hypothesis testing task [31]. The Kolmogorov-Smirnov test is also used to determine if the distribution of scores was normal or not. Then, a number of inferential statistical methods including Analysis of Variance (One-way ANOVA), Chi-square Test, Kruskal-Wallis and the Factorial ANOVA will be used to test hypotheses and draw conclusions from the data. One-way ANOVA is used to determine whether there are any significant differences in the reproductive practices of Iranian women between their various patterns of marriage. More importantly, to measure whether a combination of independent and confounding variables (e.g. the socio-demographic characteristics and more importantly the patterns of marriage) predicts the value of a reproductive practice, and to manage the effects of the confounding factors, applicable designs of the Factorial ANOVA will be used.

### Phase two: qualitative study

The second phase of this study, the qualitative research, is expected to examine reproductive practices of Iranian women by the patterns of marriage. The qualitative study is then undertaken in order to interpret or reflect on the quantitative findings in terms of deeper understanding of what meanings and reasoning these women of reproductive age attribute to their reproductive practices regarding the patterns of marriage.

#### Sample size and sampling strategy

As widely recognized in the literature, there are no rules for sample size in qualitative research. Sample size could not be determined by a prior qualitative research. Rather, sampling continues until data saturation is reached. The sampling approach in this phase will be purposeful (qualitative sampling) and the participants will be recruited based on the quantitative results. This means that, addressing the study questions (interview guide) and recruitment of participants for qualitative phase will be conducted on the basis of the primary quantitative results. This design is a kind of emergent approach in the MMs studies [24].

#### Data collection

Data are collected or made through individual semi-structured interviews. After inviting the recruited women to participate in these interviews, the time and location of the interviews will be determined at their convenience. Prior to conducting the interviews, the research team reviews the questions, and the ways to obtain valid and rich data, focusing on research questions. After obtaining informed consent, the individual interviews will be carried out in Persian or the Mazandarani tongue at the participant’s convenience. These interviews will be continued until data saturation is achieved, that is, no new themes, reasoning or explanations emerge. Along with the interviewer notes in the course of the interviews, each interview is audio-recorded. The interviewer is a PhD student of reproductive health with remarkable experience and educational background in midwifery. She is the principal researcher of this MMs study and the corresponding author of this manuscript. She was trained in qualitative interviewing as part of her doctoral training.

#### Data analysis

In this MMs study, the qualitative data are analyzed to ensure the better explanation and interpretation of the quantitative findings. This procedure is conducted using a conventional content analysis approach. The research team reviews the interviews, and extracts codes, themes and categories in order to evaluate the accuracy of the entire procedure. As Hsieh and Shannon (2005) [32] described, of three approaches to the qualitative content analysis, the advantage of the conventional one is gaining direct information from study participants without imposing preconceived categories and previous theoretical perspectives. In this way, knowledge produced from the content analysis is based on the unique understandings or interpretations of the participants and is rooted in the text data or the interviews.

In this part of the research, the recordings are listened to several times in order to form a general scheme. After this, each audio-taped interview was exactly transcribed by the corresponding author of this manuscript and read again and again in order to receive its apparent and inner elements. In this point, each written word and phrase is considered as a unit of analysis. Concurrently re-studying the manuscripts and commentary notes, will help to diagnose the initial communications among the notions extracted from the women’s statements. The text is coded and codes are iteratively put in sentences or important paragraphs until the categories and themes of women of reproductive age finally merge. It is expected that findings and themes of this phase will provide further explanation and interpretation of the quantitative survey findings.

A computer-assisted program, namely MAXQDA, version 10, is used to facilitate the data management and coding process. In the end, the research team during analysis of qualitative data will determine themes and categories that are proponents of the results achieved from the first phase of this study.

## Results: integration of quantitative and qualitative evidence

**Fig. 1:**
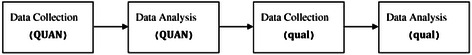
Visual model of the explanatory sequential mixed methods study: Initially, the primary quantitative phase with greater weight and emphasis will be implemented and followed by qualitative phase with lower weight and emphasis regarding the result of this stage

As noted by Andrew and Halcomb (2009), designing and doing MMs study goes beyond just collecting and analyzing quantitative and qualitative data. Rather, these different aspects require integration to develop a more comprehensive picture of the results than when they are implemented alone. Hence, at this stage of the study, besides the systematic review of the related literature on the reproductive practices of women of reproductive age and determinants and associates of these practices including the patterns of marriage, the integration of quantitative and qualitative results will be performed in practice to achieve the objectives of the study. The visual presentation of the procedures in this MMs study is shown in Fig. 1 and Table 1.

**Table 1:** Diagram for this MMs study with the explanatory sequential design: following carrying out and analysis of quantitative data in the first phase, the second phase of the study, namely the qualitative research, will be designed and conducted for the purpose of further interpreting the quantitative findings. As a final point, the integration of quantitative and qualitative results will be performed to achieve a more comprehensive picture of the results and make them more sensible and interpretable

Phase	Procedure	Product
Collecting the quantitative data	Cross-sectional study (N≈ 850)	Numeric data
Quantitative data analysis	Exploratory Data Analysis and data screening (frequencies, percent, univariate, multivariate) using IBM SPSS software V.22	Descriptive statistics, linearity, multivariate outliers, hypothesis testing
Case selection: Development of the Interview Protocol	Purposeful recruitment based on typical response and maximal variation principle	Case (?)
	Developing interview questions	Interview protocol
Collecting or making the quantitative data	Individual in-depth interview with participants (Until data saturation)	Text (non-numeric) data (interview transcripts)
Qualitative data analysis	Coding and thematic analysis	Text data (interview transcripts)
	Development of the within- case and across-case themes using the constant comparative methods (e.g. conventional qualitative content analysis)	Codes, themes and categories
		Similarity and dissimilarity of codes, themes and categories
Integration of the Qualitative and the Quantitative Results	Interpretation and explanation of the quantitative and qualitative results simultaneously	Discussion
		Implications for the policy and the public
		Future research

### Ethical consideration

In both phases of this MMs study, the written informed consent will be obtained from each participant. Permission to carry out this study and its design was obtained from The Ethical Committee of Tehran University of Medical Sciences (TUMS), Tehran, Iran on 22 March 2013 (Ref: 92–130–1297).

## Discussion

This MMs study is to explain the main reproductive practices of Iranian women living in Babol city, Iran using the determinants of fertility with a focus on the patterns of marriage. It provides, in fact, a fresh response to a comparatively conventional question in the literature, namely ‘the patterns of marriage and reproductive practices’ in the socio-cultural and political context of contemporary Iran. This study is developed to respond to the increasing demand of reconsidering the population and family planning policy in the country, due to significant transformations in the reproductive practices of Iranian people during the last two decades [33]. Therefore, there are contesting solutions for the questions like what kind of population policy should Iran have these days and what are more important determinants of reproductive practices in the country?

In this view, as marriage and its patterns potentially have implications for the status of women, their health and fertility, monitoring marriage patterns and dynamics can contribute to answering the aforementioned questions precisely. It is supposed that the results of this study can play an important role henceforth for designing and planning health-related science-based interventions needed to reform women’s reproductive practices and promote their health long-term. As a number of researchers observed, reproduction varies in line with the pattern of marriage and this difference is mainly created by the characteristics of socioeconomic diversity which are associated with the differences in the type of mate selection [34]. A closer look at the existing body of literature revealed that the effect of patterns of marriage on the reproductive practices is not the same. While some studies showed fertility rate in people with traditional patterns of marriage is higher than those married in modern style [35, 36], other studies reported different results [13, 34, 37–39]. One limitation of this study is convenience sampling in the last cluster, because of the limitations of random sampling.

As reproductive practices are central to the sexual and reproductive health of women, studying the determinants of these practices including patterns of marriage helps the policymakers and health promoters in creating appreciate agendas and policies to promote health of women and children, subsequently, the health of their families in Iran and those of other women who may have similar beliefs, habitudes and practices that need to be dealt with effectively. Collecting both quantitative and qualitative data and integrating all objective and subjective findings enables a better understanding of the research objectives. As a result, further study in this area, particularly in the developing countries like Iran is required. The findings of this MMs study may help public health educators, demographers, women’s health activist and policy makers to better understand the critical role of patterns of marriage in reproductive practices.

## Abbreviations

QUAN: Quantitative
qual: Qualitative
SPSS: Statistical Package for Social Science
PPS: Probability Proportional to Size
MMs: Mixed Methods
TPB: Theory of planned behavior
EDA: Exploratory Data Analysis
